# Ultrasonography-Guided Core Biopsy of Supraclavicular Lymph Nodes for Diagnosis of Metastasis and Identification of Epidermal Growth Factor Receptor (EGFR) Mutation in Advanced Lung Cancer: Erratum

**DOI:** 10.1097/01.md.0000471207.95605.fb

**Published:** 2015-08-21

In the article “Ultrasonography-Guided Core Biopsy of Supraclavicular Lymph Nodes for Diagnosis of Metastasis and Identification of Epidermal Growth Factor Receptor (EGFR) Mutation in Advanced Lung Cancer”,^1^ which appeared in Volume 94, Issue 29 of *Medicine*, Figure 1 contained a typo. Also, in the second sentence of the legend for Figure 5, a “greater than” sign should have been captured as a “greater than or equal to” sign. The sentence should have read “Patient who has an enlarged SCN visible on initial chest CT ≥0.85 cm in the short-axis dimension undergo US-guided core biopsy of SCN”. The article has since been corrected online.
